# Congestive heart failure after enterotomy in a cat with asymptomatic transient myocardial thickening

**DOI:** 10.1186/s13620-025-00312-4

**Published:** 2025-10-24

**Authors:** Sin-Wook Park, Keon Kim, Young-Jae Lee, Yoon-Jung Do, Woong-Bin Ro, Chang-Min Lee

**Affiliations:** 1 Department of Veterinary Internal Medicine, College of Veterinary Medicine and BK21 FOUR program, Chonnam National University, Gwangju, Republic of Korea; 2 Re-born Animal Medical Centre, Busan, Republic of Korea; 3 Animal Diseases & Health Division, Rural Development Administration, National Institute of Animal Science, Wanju-gun, Republic of Korea; 4 College of Veterinary Medicine, Chonnam National University, Gwangju, 61186 Republic of Korea

**Keywords:** Feline, Heart disease, Myocardial disease, Subclinical

## Abstract

Transient myocardial thickening (TMT) is characterised by reversible left ventricular myocardial thickening. A 2-year-old castrated male British Shorthair was presented with a history of severe vomiting for 2 days. Based on abdominal radiography and ultrasonography, the cat was diagnosed with an obstructive gastrointestinal foreign body. Preoperative echocardiography revealed an increased maximum left ventricular wall thickness (LVWT: 6.9 mm, measured at end-diastole) and ratio of the left atrium to the aortic root (LA/Ao: 2.1), indicative of a hypertrophic cardiomyopathy phenotype. An enterotomy was performed, and the foreign body was found to be an almond. Immediately after surgery, the cat became tachypnoeic, and thoracic radiography revealed pulmonary oedema. The cat was then treated with cardiac medications. Five days after surgery, the cat’s condition clinically normalised. Two months after the first presentation, repeated echocardiography revealed a decreased LVWT (4.8 mm) and LA/Ao (1.58). The cat was diagnosed with TMT, and all cardiac medications were discontinued. The cat remained clinically well for 14 months after the last presentation. This is the first case report to demonstrate that foreign body ingestion may cause TMT, and that congestive heart failure (CHF) can develop after enterotomy in cats with subclinical TMT. The possibility of TMT should be considered in cats with foreign body ingestion that requires anaesthesia and/or surgery because it may trigger CHF, which could ultimately lead to death.

## Introduction

Transient myocardial thickening (TMT) has been recently described in cats and is characterised by reversible left ventricular (LV) myocardial thickening [1, 2]. TMT has been reported to potentially cause left atrial (LA) enlargement, congestive heart failure (CHF), and arterial thromboembolism [1, 3]. Unlike the progressive degradation of cardiac function seen in hypertrophic cardiomyopathy (HCM), improved cardiac function can occur in TMT, making it reversible and leading to a better prognosis [1, 4].

The exact aetiology of TMT is unknown in cats, but previous studies have hypothesised that TMT may be manifestations of myocarditis of various causes or possibly stress-induced myocardial dysfunction as reported in humans [4]. The most common preceding events related to feline TMT are surgical/anaesthetic factors, but other events include infections (e.g., bartonellosis, toxoplasmosis), traffic accidents, and thermal burns [1, 2, 5–7].

To the author’s knowledge, TMT is well demonstrated in cats, but there are no reports that describe the development of CHF in cats with asymptomatic TMT after surgery. This report describes a cat that developed asymptomatic TMT after foreign body ingestion and developed CHF after an enterotomy. This cat required urgent abdominal surgery, and anaesthetic and post-operative considerations are also described.

## Case description

A 2-year-old castrated male British Shorthair weighing 3.4 kg was referred to the animal medical centre with a history of severe vomiting for 2 days. The cat was a fully vaccinated, indoor-only cat and had no prior medical history. Due to the possibility of dehydration, the cat was given intravenous (IV) fluids and an antiemetic (maropitant 1 mg/kg IV) before the referral. On physical examination at presentation, the cat’s body temperature was 38.3 °C, a regular heart rate (138 beats/min) with normal heart sounds was detected, the cat’s systolic blood pressure was normal (130 mmHg), and no remarkable dehydration was detected. Abdominal radiography in ventral and right lateral recumbency revealed an enlarged stomach and dilated small intestines. Abdominal ultrasound revealed two centimetres of hyperechoic material with distal acoustic shadowing in the jejunum and proximal small intestine dilation. No remarkable findings were found on a complete blood count and blood chemistry (creatinine: 97 µmol/L, reference range: 70–212 µmol/L; blood urea nitrogen, BUN: 10 mmol/L; reference range: 6–13 mmol/L), and a blood gas analysis revealed a metabolic alkalosis (pH = 7.452, reference range: 7.21–7.41; pCO_2_ = 46 mmHg, reference range: 28–50 mmHg; HCO_3_ = 32.2 mmol/L, reference range: 21–28 mmol/L), hypokalaemia (2.7 mmol/L; reference range: 3.5–5.5 mmol/L), hyperchloremia (107 mmol/L; reference range: 113–128 mmol/L). The cat was diagnosed with foreign body ingestion and received IV fluids (500 ml of saline with 10 mmol of KCl, 3 ml/kg/h), metronidazole (10 mg/kg IV), and maropitant (1 mg/kg IV). Preoperative fasting was advised for 12 h prior to surgical intervention. Elective surgery was planned for the next day.

**Fig. 1:**
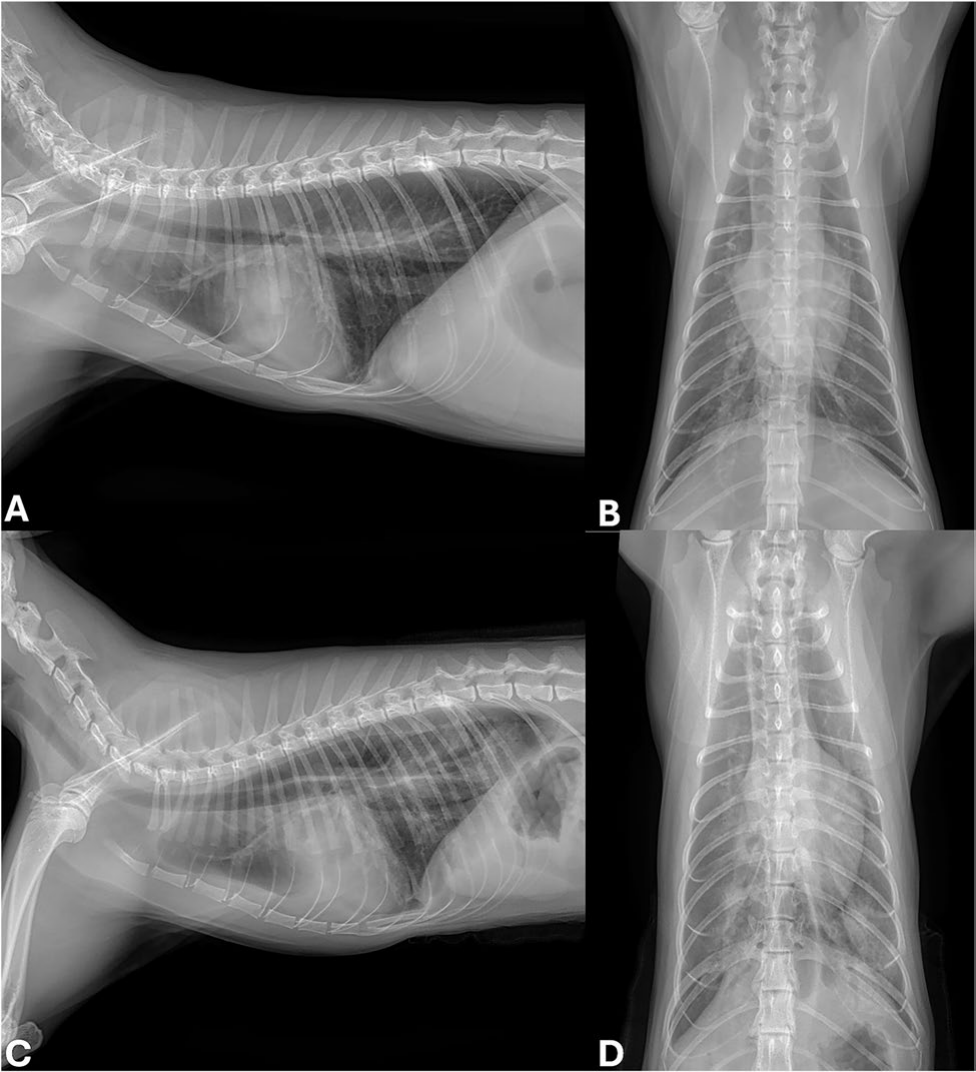
Right lateral (**A**, **C**) and ventrodorsal thoracic radiographs (**B**, **D**) taken before (**A**, **B**) and after enterotomy **C**, **D**. Considerable alveolar infiltration from the perihilar region to the caudal lung lobes suggests that congestive heart failure had developed following enterotomy **C**, **D**

**Fig. 2:**
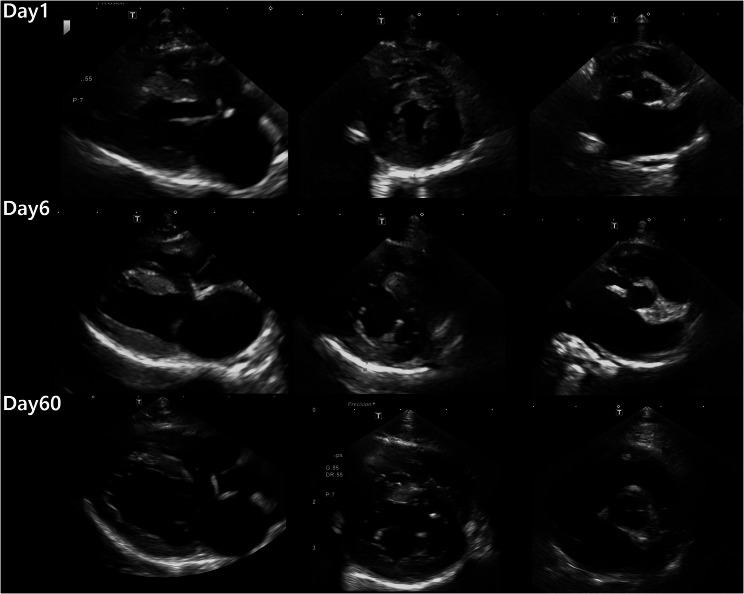
Two-dimensional echocardiographic images of right parasternal views at three timepoints. From the left, a right parasternal long-axis four-chamber view, right parasternal short-axis view at the papillary muscle level, and a right parasternal short-axis view at the aortic valve level are shown. The left ventricular wall thickness (LVWT) was measured from the thickest segment using two-dimensional imaging of the right parasternal long-axis four- and five-chamber views and the short-axis view at the papillary muscle level. Note the significant decreases in LVWT and left atrial diameter on day 60

Twelve hours later, preoperative thoracic radiography was performed to evaluate tracheal diameter for endotracheal tube selection and to screen for any cardiopulmonary abnormalities prior to general anaesthesia. Radiographs in ventral and right lateral recumbency showed an increased cardiothoracic ratio, dilation of the pulmonary artery, and a mild unstructured interstitial lung pattern (Fig. 1). Echocardiography was performed by one veterinary radiologist and analysed retrospectively by a board-certified internist (Table 1). Symmetrical hypertrophy of the LV wall, with a maximum left ventricular wall thickness (LVWT: 6.9 mm), an increased left atrium to aortic root ratio (LA/Ao: 2.1), and complete fusion of early and late filling waves (48 cm/s) were observed (Fig. 2; Table 1). Electrocardiography revealed sinus tachycardia with a heart rate of 180 beats per minute and a normal QRS configuration. The cat was diagnosed with an HCM phenotype. The cat was premedicated with cefazolin (20 mg/kg IV), maropitant (1 mg/kg IV), butorphanol (0.2 mg/kg IV), midazolam (0.2 mg/kg IV), and furosemide (2 mg/kg IV). Premedication with butorphanol and midazolam was selected to minimise cardiopulmonary depression. Intravenous fluid therapy initiated prior to surgery continued throughout the anaesthetic period. Anaesthesia was induced by an IV injection of propofol (5 mg/kg IV) and was maintained by the administration of isoflurane. Oxygen saturation (SpO₂) and blood pressure were continuously monitored throughout anaesthesia, and no abnormalities were observed. The total anaesthetic duration from induction to recovery was approximately 55 min. Following an incision made through the skin, subcutaneous tissue, and linea alba, the obstructed intestinal loop was exteriorized. On the antimesenteric border and distal segment of the obstruction, a linear incision was made to remove the foreign body, which was found to be an almond. The enterotomy and surgical wounds were closed routinely in layers using standard suture techniques. Postoperative thoracic radiography was preplanned to screen for potential anaesthesia-associated cardiopulmonary complications, given the echocardiographic findings consistent with an HCM phenotype. Radiographs in ventral and right lateral recumbency revealed pulmonary oedema in the left caudal lung lobe (Fig. 1). The previous fluid therapy was discontinued, and a solution of dobutamine diluted in 0.9% sodium chloride was continuously administered at a rate of 3 mL/h (3 ug/kg/min) due to a decrease in systolic blood pressure to 70 mmHg. The cat received oxygen supplementation, cefazolin (20 mg/kg IV BID), metronidazole (10 mg/kg IV BID), marbofloxacin (3 mg/kg IV SID), maropitant (1 mg/kg IV SID), butorphanol (0.2 mg/kg IV TID), dalteparin (150 IU/kg TID), and furosemide (1 mg/kg IV q4h).

**Table 1:** Selective two-dimensional echocardiographic variables on echocardiographic examinations after the first presentation (Day 0)

Variable	Units	Day 1	Day 6	Day 60
Heart rate	beat/min	180	160	168
Blood pressure	mmHg	90	110	120
LAD max	mm	15.3	16.1	11.4
LA/Ao		2.1	2.49	1.58
LVIDd	mm	10.2	9.5	12.7
LV FS	%	69	65	64
LVWT Max	mm	6.9	6.5	4.8
IVSd MAX (RPLax)	mm	6.2	6.5	4.8
LVFWd MAX(RPLax)	mm	6	6.2	4.2
IVSd MAX (RPSax)	mm	6.9	6.5	4.6
LVFWd MAX(RPSax)	mm	5.9	6.3	4.3
AV Vmax	cm/s	85	-	82
PV V max	cm/s	40	-	92

*LAD Max* Maximal left atrial diameter, *LA/Ao* Left atrium to aortic root ratio, *LVIDd* Left ventricular internal diameter in end-diastole, *LV FS* Left ventricular fractional shortening, *LVWT Max* Maximal left ventricular wall thickness, *IVSd Max* Maximal interventricular septal thickness in end-diastole, *LVFWd Max* Maximal left ventricular free wall thickness in end-diastole, *AV Vmax* Peak velocity across the aortic valve, *PV Vmax* Peak velocity across the pulmonary valve, *RPLax4C* Right parasternal long-axis four-chamber view, *RPLax5C* Right parasternal long-axis five-chamber view, *RPSax* Right parasternal short-axis view. Echocardiographic measurements were performed by one radiologist and analysed retrospectively by a single author. No sedation was used for each echocardiogram

The following day, the cat’s systolic blood pressure increased to 90 mmHg, and thoracic radiography showed an increased alveolar pattern, which was accompanied by an increased respiratory rate (36 to 54 breaths/min). On blood chemistry tests, a high creatinine (239 µmol/L) and BUN (19 mmol/L) were identified. As the owner requested discharge, pimobendan (0.3 mg/kg BID), clopidogrel (18.75 mg SID), furosemide (2 mg/kg BID), spironolactone (1 mg/kg BID), famotidine (0.5 mg/kg BID), and metronidazole (10 mg/kg BID) were prescribed, and the cat was discharged.

Four days after discharge, the owner reported improvements in the cat’s overall condition. Thoracic radiography showed significantly decreased pulmonary oedema. An echocardiographic examination revealed hypertrophy of the LV wall (LVWT maximum: 6.5 mm) and increased LA/Ao (2.49) (Fig. 2). On blood chemistry tests, normalised plasma creatinine (115 µmol/L) and BUN levels (12 mmol/L) were revealed. The medical treatment was maintained, and follow-up was recommended at the local vet clinic.

Two months after the first presentation, the cat was presented for repeat echocardiography. Echocardiography revealed a reduced LVWT (LVWT maximum: 4.8 mm) and LA/Ao (1.58); the peak velocity of early diastolic transmitral flow was 58 cm/s, and the peak velocity of late diastolic transmitral flow was 60 cm/s (Fig. 2). The cat was diagnosed with TMT, and all cardiac medications were discontinued. Fourteen months after the first presentation, the owner reported by telephone that the cat was doing clinically well.

## Discussion

CHF may develop following anaesthesia or surgery in cats with primary HCM [8]. However, to the best of our knowledge, the development of CHF in a cat with asymptomatic TMT following surgery has not been previously described. Asymptomatic TMT may be underreported because affected cats typically do not show clinical signs and the myocardial thickening often resolves spontaneously within weeks to months without detection [1, 2]. Therefore, asymptomatic TMT may not require treatment. However, caution is necessary for cats with asymptomatic TMT undergoing anaesthesia or surgery, as they may develop CHF, which could ultimately lead to death. Consequently, preoperative echocardiography may be crucial for prognostic assessment in cats with underlying diseases requiring anaesthesia or surgery.

There are reports of TMT being potentially associated with antecedent events including anaesthesia, surgery, and road traffic accidents as well as various diseases and conditions including thermal burn injuries, and infectious diseases such as toxoplasmosis, bartonellosis, and sepsis [1, 2, 5–7]. In addition, TMT is more common in young cats than HCM [1]. This study is the first to confirm TMT after foreign body ingestion in a cat. The development of TMT in this case may be related to the emotional stress caused by severe vomiting induced by foreign body ingestion [2, 9]. Since foreign body ingestion is more common in young cats, the possibility of subclinical TMT should be considered in cats with foreign body ingestion that require anaesthesia and/or surgery [10].

Recent studies have defined TMT by identifying an initially elevated LVWT (≥ 6 mm) followed by a decreased LVWT (< 5.5 mm) on follow-up echocardiography [1]. In this cat, the LVWT initially measured 6.9 mm but thinned by approximately 30% to 4.8 mm after two months, meeting the diagnostic criteria for TMT. In addition, the LA diameter, which was 15.3 mm during the asymptomatic phase, increased to 16.1 mm after development of CHF and later decreased to 11.4 mm after normalisation of myocardial thickness. This suggests that the LA diameter may be associated with the LVWT, which may affect cardiac function, and the LA/Ao may be significantly enlarged in cats with asymptomatic TMT [11, 12].

Anaesthesia, surgery, IV fluid administration, and potentially corticosteroid administration may lead to CHF in cats with subclinical HCM [11, 13]. Acute CHF occurs occasionally in human patients with HCM undergoing noncardiac surgery and is probably related to diastolic dysfunction [14]. In cats with HCM, tachycardia shortens the duration of diastole and therefore limits coronary perfusion, potentially leading to worsen diastolic dysfunction and precipitating CHF [13]. There is no report of anaesthetic consideration of cats with TMT. In the present case, the cat was assumed to have HCM at the time of surgery, and the anaesthesia was planned accordingly. Despite this, the cat developed CHF during the hospitalisation period, which is a potential consequence of general anaesthesia or surgery in cats with significant heart disease. In the present case, the progression of previously asymptomatic TMT to CHF during hospitalisation may be explained by the intravenous fluid administration, general anaesthesia, and natural progression of TMT.

Treatment of clinical TMT is similar to that for HCM in cats and includes diuretics [1, 2]. Furthermore, there is a report of feline arterial thromboembolism in TMT, suggesting that antiplatelet therapy, such as clopidogrel, may be necessary [3]. In contrast to HCM, which is a progressive disease requiring lifelong treatment, TMT is characterised by spontaneous resolution of LV wall thickening within a few months, and the prognosis remains favourable with discontinuation of cardiac medication [1]. In this cat, normalisation of myocardial thickness and LA diameter were observed after two months. However, other reports have documented cases where myocardial normalisation occurred within one month [9, 15]. Thus, in suspected cases of TMT, repeated echocardiography may help determine whether discontinuation of cardiac medication can be attempted in cats that no longer require treatment.

The main limitation of this report is that diagnostic tests for specific infectious diseases were not performed. However, this cat was a strictly indoor-only cat, which made systemic infection less likely [9]. In addition, since fluid therapy was administered before the initial echocardiographic examination, the cardiac parameters may have been affected. In addition, cardiac injury markers such as troponin I were not measured.

In conclusion, a two-year-old cat with asymptomatic TMT following obstructive foreign body ingestion developed CHF after undergoing an enterotomy. The possibility of TMT should be considered in cats with foreign body ingestion that requires anaesthesia and/or surgery because it may trigger CHF, which could ultimately lead to death.

## Data Availability

No datasets were generated or analysed during the current study.

## Abbreviations

BUN: Blood urea nitrogen
CHF: Congestive heart failure
HCM: Hypertrophic cardiomyopathy
HCO_3_: Bicarbonate
IV: Intravenous
IVSd: Interventricular septal thickness in diastole
LA: Left atrial
LA/Ao: Left atrium to aortic root ratio
LAD max: Maximum left atrial diameter
LV: Left ventricular
LVIDd: Left ventricular internal diameter in diastole
LVFS: Left ventricular fractional shortening
LVFWd: Left ventricular free wall thickness in diastole
LVWT: Left ventricular wall thickness
RPLax: Right parasternal long axis
RPSax: Right parasternal short axis
TMT: Transient myocardial thickening
